# Outcomes of Birmingham Hip Resurfacing Based on Clinical Aspects and Retrieval Analysis of Failed Prosthesis

**DOI:** 10.3390/ma17163965

**Published:** 2024-08-09

**Authors:** Iulian Antoniac, Niculae Valeanu, Marius Niculescu, Aurora Antoniac, Alina Robu, Larisa Popescu, Veronica Manescu (Paltanea), Dan Anusca, Catalin Ionel Enachescu

**Affiliations:** 1 Faculty of Material Science and Engineering, National University of Science and Technology Politehnica Bucharest, 313 Splaiul Independentei, District 6, RO-060042 Bucharest, Romania; antoniac.iulian@gmail.com (I.A.); niculae.valeanu@stud.sim.upb.ro (N.V.); alinarobu2021@gmail.com (A.R.); maria.popescu2010@stud.sim.upb.ro (L.P.); veronica.paltanea@upb.ro (V.M.); 2 Academy of Romania Scientists, 54 Splaiul Independentei, RO-050094 Bucharest, Romania; 3 Faculty of Medicine, Titu Maiorescu University, 67A Gheorghe Petrascu, RO-031593 Bucharest, Romania; mariusniculescu1961@gmail.com; 4 Department of Orthopedics and Trauma I, Colentina Clinical Hospital, 19-21 Soseaua Stefan cel Mare, RO-020125 Bucharest, Romania; 5 Faculty of Electrical Engineering, National University of Science and Technology Politehnica Bucharest, 313 Splaiul Independentei, District 6, RO-060042 Bucharest, Romania; 6 Department of Orthopedics, University of Medicine and Pharmacy of Craiova, 2-4 Petru Rares, RO-200349 Craiova, Romania; drdananusca@yahoo.com; 7 Department of Dermatology, Elias Emergency University Hospital, 17 Bulevardul Marasti, RO-011461 Bucharest, Romania; catalin_enachescu@yahoo.com

**Keywords:** BHR prosthesis, bone cement, cement technique, metallic biomaterials, retrieval analyses

## Abstract

This research aims to identify the prevalence of failure for Birmingham Hip Prosthesis (BHR) in total hip arthroplasty and to analyze its reasons from biomaterials and biofunctional perspectives. We present our current analysis and tests on a series of different BHR-retrieved prostheses after premature failure. Relevant clinical data, such as X-ray investigations and intraoperative images for clinical case studies, were analyzed to better understand all factors involved in BHR prosthesis failure. A detailed analysis of the failures highlighted uneven cement distribution, overloading in certain areas, and void formation in the material. A closer investigation using microscopical techniques revealed the presence of a crack originating from the gap between the cement mantle and human bone. Additionally, scanning electron microscopy analyses were conducted as part of the investigation to examine bone cement morphology in detail and better understand the interactions at the interfaces between implant, cement, and bone. In conclusion, this research emphasizes the importance of surgical technique planning and the cementation procedure in the success rate of BHR prostheses. It also underscores the need to carefully evaluate patient characteristics and bone quality to minimize the risk of BHR prosthesis failure. The cementation procedure seems to be essential for the long-term functionality of the BHR prosthesis.

## 1. Introduction

Birmingham Hip Resurfacing (BHR) arthroplasty is an option in hip replacement surgery with a different approach to classical total replacement [1,2,3,4]. During the procedure, instead of completely replacing the femoral head and acetabular cavity with artificial components, BHR prostheses aim to preserve healthy bone as much as possible [5,6]. Thus, only the articulated surface of the femoral head is replaced by a metal component, while the acetabulum is preserved and covered with another metal component. Younger and more active patients especially prefer this technique, as it allows them to maintain a broader range of motion and avoid certain limitations associated with total hip replacement [7,8,9,10].

In the case of old medical techniques used in BHR prostheses, the studies did not lead to favorable results due to inflammatory reactions and wear particles that formed, which ultimately led to the mobilization of the prosthesis into the bone [11,12,13,14,15,16,17,18,19]. As required by the BHR prosthesis protocol, the mobilization and implantation of the prosthesis are crucial to its success, especially in the case of new BHR technologies that lack a femoral component that gives a higher degree of stability. Thus, the insertion angle has to be chosen precisely, and the prosthesis must be introduced by considering that no dysfunctions due to biomechanics will occur [20]. At the same time, the use of the appropriate cement and its adequate insertion into the acetabular head plays an important role in the longevity of the implant [21,22,23,24,25,26,27]. Modern methods of cementing involve using a special plug adapted to the shape of the bone, which can be made of cement or centromedular plastic. This plug is designed to fit tightly to the femoral canal, and during the surgical procedure, a pressure lavage-suction system is used to clean it from all remnants of the bone [28,29]. Bone cements do not have adhesive properties but rely on the mechanical connection between the bone surface and the prosthesis. They play an important role as critical media for securing implants and facilitating healing. Understanding these interactions is vital for improving surgical outcomes and enhancing the longevity of orthopedic implants. The cement formulation is based on a homogeneous mixture between a powder and a liquid phase [30]. The typical setting involves mixing the polymer powder and liquid monomer, leading to a chemical reaction forming a hardened structure. This exothermic process releases heat that can affect surrounding tissues and implants. The obtained material has special flexibility and modeling, which ensures fixation at the implant site and good bone-biomaterial contact. Cement based on poly(methyl methacrylate) (PMMA) is frequently used in orthopedic practice. The formulation of PMMA bone cement typically includes a polymer powder composed mainly of PMMA or copolymers of PMMA with other acrylates that may also contain radiopacifiers such as barium sulfate or zirconium dioxide to make the cement visible under X-rays and a liquid monomer (methylmethacrylate (MMA) monomer), which polymerizes to form a solid matrix. In addition, the liquid may contain stabilizers like hydroquinone to prevent premature polymerization and initiators like benzoyl peroxide to start the polymerization process. Antibiotics, essential oils, or silver nanoparticles [26] may be added to prevent an infection from spreading after surgery, to manage an existing illness, or to shield medical equipment from bacterial colonization [31]. Additionally, plasticizers and crosslinking agents can be included to modify the cement’s mechanical properties and setting characteristics. Table 1 presents the cement behavior in relationship to bone structure [32,33,34,35].

BHR-type prostheses, with short tails and metal components, can be beneficial for young people with high bone rigidity and capacity for new tissue formation, producing the phenomenon of osseointegration in an accelerated time, and prosthesis fixation can be easily achieved. Unfortunately, in the case of older patients, it is not advised to use such types of prostheses because decreased bone density is usually related to implant migration and metallosis [37,38,39,40].

There are some important studies in the literature regarding the outcomes and failure analyses of BHR arthroplasty. Molloy et al. [41] recently investigated the long-term outcomes of BHR by performing a systematic review, in which they included independent case reports with a minimum follow-up time of about 10 years. They found 11 studies that included 3129 cases. A number of 9 papers estimated a mean follow-up of about 11.7 years, with a pooled 10-year survival rate of 95.5%. The authors identified 149 revisions, which represented a percentage of 4.8% of the investigated studies. It was observed that the main failure cases were due to aseptic loosening and side effects generated by metallic particles and debris, representing 20.1% for each cause of the total number of revisions. The main conclusion of this study was that BHR prostheses could offer patients a minimum implant life of about 10 years if the surgeon’s indications are strictly respected. In addition, a low number of infections associated with hip arthroplasty was reported in the study, a fact that proved that BHR prostheses are a safe option and offer good functional results. Another interesting statistical analysis performed by Lass et al. [42] took into account the 5-year results of Birmingham hip resurfacing arthroplasty performed by only one surgeon between 2007 and 2012 on 154 patients. The authors divided the patients (18 women/20 hips and 136 men/163 hips) into low-risk (149 with a size larger than 48 mm of the femoral component) and high-risk cases (34 characterized by a femoral head with a geometrical size below 48 mm). It was possible to follow up only 91% of the human subjects over 5 years. An overall survival rate of the prostheses based on the Kaplan-Meier analysis was established to be equal to 91.8% (15 revisions). In the low-risk patient group, the free survival rate was 96.6%, characterized by only 5 revisions. Regarding these 5 revisions, the main surgical causes were: 1 patient with an unseated cup that generated a longer leg and pain, 2 patients with femoral component loosening and increased metallic ion levels, 2 patients with osteolysis and increased levels of cobalt (Co) and chromium (Cr) at about 53 μg/L and 71 μg/L, respectively. In the high-risk group, the survival rate was 70.6% (10 revisions). The revisions were generated by femoral component loosening (2 patients), pain (1 patient), and high concentrations of metallic ions (7 patients). It was demonstrated that BHR prostheses exhibited a very good mid-term survival. In addition, Birmingham hip resurfacing is an adequate surgical procedure for patients with a femoral head size higher than 48 mm. Uemura et al. [43] investigated the long-term results of Birmingham hip resurfacing arthroplasty in the case of Asian patients. In this cohort, an increased number of hip dysplasia and osteonecrosis are present and supplementary; the females had a small femoral head. A number of 112 Japanese patients (59 females and 53 males/130 hips) who underwent a BHR arthroplasty were selected for this study. The average age was 52, and the follow-up time was 12 years. The overall survival rate was found to be 96.5% (6 revisions). The main revision causes were femoral component aseptic loosening (2 patients), infection (2 patients), cup aseptic loosening (1 patient), and femoral neck fracture (1 patient). By eliminating the septic revision, the implant survival rate was equal to 98.2% at 10 years. The authors did not find a direct relationship between patient sex, hip disease particularities, femoral head size, and implant survival time and concluded that in the case of the Japanese cohort, the BHR prostheses are adequate, and Asian people’s particular features do not influence their function. Frew and Johnson [44] chose the particular case of young men (19 ÷ 55 years old) with BHR prostheses and performed a follow-up of about 13 years. The authors analyzed 155 patients/147 hips. A number of 11 secondary arthroplasty surgeries were performed, and an overall cumulative survival rate of 88.8% at 13 years was computed. It was concluded that BHR prostheses remain a valid option for young male patients, but the surgeons recommended that alternative bearings should be considered in some cases. Matharu et al. [45] studied the prevalence of femoral neck fracture after BHR arthroplasty. They selected 3076 patients from the same hospital and observed that fractures were present in 34 hips, determining a prevalence of 1.1% and a median time to fracture of 0.27 years. At a follow-up time of 5.5 only 3 patients required revision surgery for aseptic loosening and sepsis. It was concluded that a survival rate of 95.7% proved that BHR prostheses are not usually linked to femoral head neck fractures.

The paper will present a retrospective study of a relevant orthopedic clinic from a Romanian hospital to identify the outcomes and reasons for the failure of BHR prostheses. The main objectives of our study are as follows:

- To identify the prevalence of failure for Birmingham Hip Prosthesis (BHR) in total hip arthroplasty;
- To analyze the reasons for BHR failure from biomaterials and biofunctional perspectives;
- To conduct a detailed analysis and tests on a series of different BHR-retrieved prostheses after premature failure;
- To analyze relevant clinical data like X-ray investigations and intraoperative images for clinical case studies to better understand all factors involved in BHR prosthesis failure;
- To investigate the importance of surgical technique planning and the cementation procedure in the success rate of BHR prostheses;
- To evaluate patient characteristics and bone quality to minimize the risk of BHR prosthesis failure.

## 2. Materials and Methods

### 2.1. The Study Group

A retrospective analysis was conducted based on data from patients admitted to Colentina Clinical Hospital in Bucharest, Romania. The study performed within the hospital took into account several specific aspects, starting from the predominant average age and the sex of patients and subsequently reaching data such as the evolution of the type of fixation of prostheses in hip resurfacing interventions and cementation of acetabular and femoral components.

**Figure 1:**
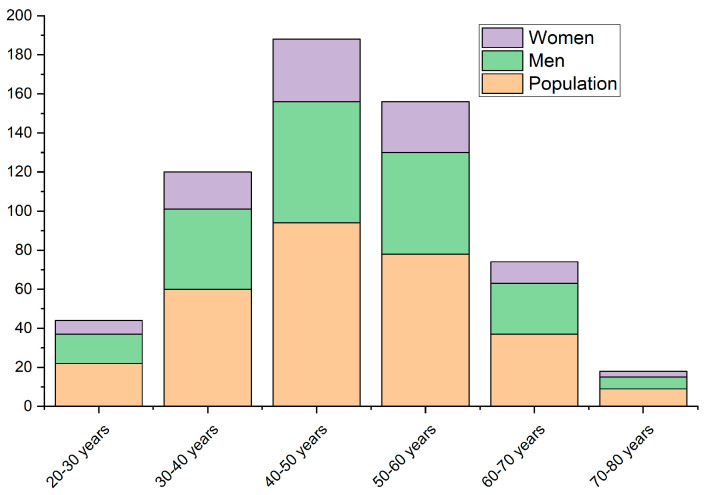
Distribution by age categories of BHR prosthesis cases from Colentina Clinical Hospital, Bucharest, Romania.

Analyzing the age distribution of hip resurfacing prosthesis cases, we notice the following results: most procedures are recorded for people aged between 40 and 60 years, suggesting a prevalence of joint diseases in this age group. In particular, in the age range of 40 ÷ 50 years, a significant number of 92 cases are noted, indicating an increased intensity of the need for surgery at this stage of life. However, cases decrease considerably in younger and older age groups. This trend may reflect, to some extent, the level of physical activity and exposure to age-related risk factors. People under 30 and over 70 years old show a lower presence in the data analyzed, suggesting a lower incidence of joint disease at younger ages or a more rigorous selection of candidates for surgery in the older age group [46]. Thus, the distribution of variations by age categories within this surgical procedure provides a complex perspective on patients’ profiles and medical needs. Figure 1 presents a distribution considering the age criteria for BHR prostheses in the analyzed cohort of patients.

During the investigation on the distribution of patients undergoing hip resurfacing prosthesis surgery in gender groups, it was found that the number of male cases was 202, while that of female cases was 98. From this comparison, it can be inferred that male patients are more likely to have orthopedic disorders requiring interventions such as hip resurfacing prosthetics compared to women [47]. However, it is important to emphasize that this inference requires a more detailed and extensive analysis to confirm specific correlations between sex and predisposition to orthopedic conditions. Table 2 shows the number of surgical cases considering the medical indication, in accordance with patient gender.

Starting from the clinical data provided and from the idea of examining the distribution of cases for different types of surgical indications according to the patient’s sex, a number of specific observations emerge. The statistical analysis of surgical cases, correlated with the patient’s gender, reveals significant differences in the distribution of surgical indications. While women are more prevalent in cases of avascular necrosis of the femoral head and primary coxarthrosis, men undergo surgery for primary coxarthrosis, avascular necrosis of the femoral head, and dysplasia. This fact suggests variations in the clinical presentation of orthopedic conditions between the sexes and the need to personalize therapeutic approaches according to individual patient characteristics. Of the 300 implants analyzed, approximately 10% of them registered failures, highlighting the continued importance of careful monitoring and rigorous evaluation of implant performance to optimize orthopedic treatment results. Overall, observations suggest that failures tended to be more common in older people, which may reflect the impact of age-related factors on implant durability and joint tissue strength.

From the statistical analysis reported in Table 3, it can be observed that the *p*-value indicates the probability that the noticeable difference between groups of men and women is purely random rather than statistically significant. In other words, the lower the *p*-value, the less likely it is that the difference is the result of chance and the more likely it is that it is statistically significant. Typically, a threshold of 0.05 is used to determine statistical significance, meaning that a *p*-value of less than 0.05 indicates a statistically significant difference between groups.

**Figure 2:**
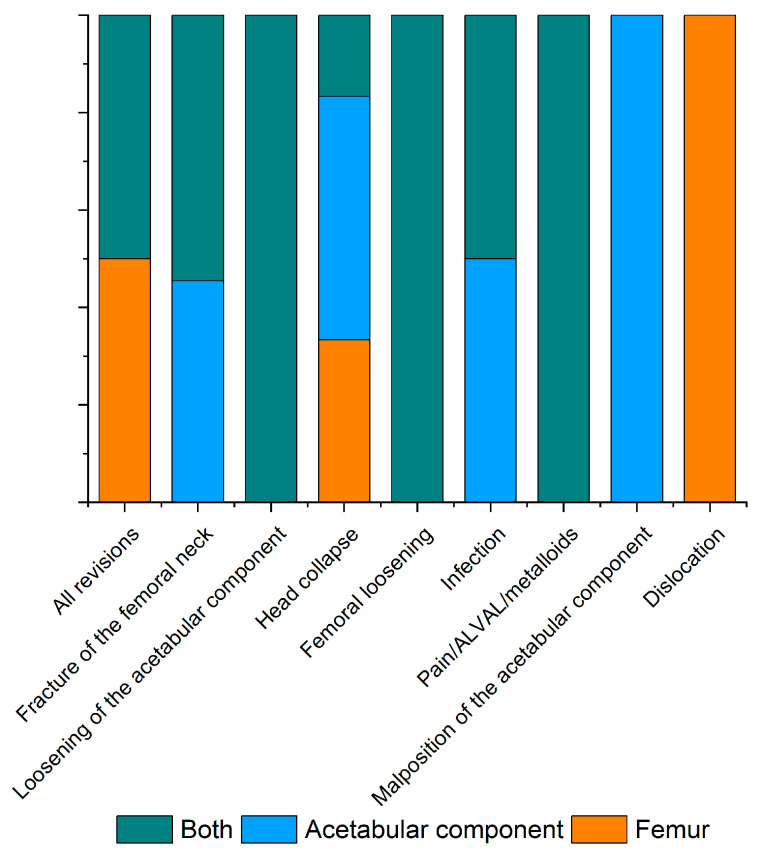
Graphical representation of failure rates in total hip resurfacing procedures (including single or both component revision surgery), with a focus on aseptic lymphocyte-dominant vasculitis-associated lesions (ALVAL).

The data presented in Figure 2 and Table 3 provide detailed insight into the failure modes of BHR implants, highlighting their distribution between men and women, along with estimated *p*-values for statistical comparisons. Of the 300 revisions, 10% were required due to implant-related issues. The most common ways of failure include fracture of the femoral neck, detachment of the acetabular component, and collapse of the femoral head. The estimated *p*-values indicate the statistical significance of gender differences for each failure mode. The lowest value of failure was obtained for “Malposition of the acetabular component”, representing 0.3% of the total revised cases, while the highest value was achieved in the case of “Fracture of the femoral neck”, representing 3% of the total revised cases. These data highlight the complexity of managing complications associated with BHR implants and the need for a personalized approach to orthopedic treatment.

Table 4 provides a comprehensive summary of pre-operative diagnoses encountered in patients requiring revision due to acetabular component loosening during total hip arthroplasty procedures. It highlights the diversity of pre-existing conditions and associated risk factors with this specific surgical complication. In the provided data, osteoarthritis is prevalent in 10 men and 5 women, without posing an adverse effect on acetabular component stability, whereas acetabular dysplasia, previous shelf procedures for acetabular dysplasia, renal failure, avascular necrosis of the femoral head following radiotherapy for pelvic osteosarcoma, and unknown diagnoses each present potential risks or uncertainties regarding acetabular component stability, with occurrences noted in both men and women.

Table 5 shows the average time to revision surgery for various failure modes after hip resurfacing procedures, comparing men and women. For example, for “Femoral neck fracture”, the average time to revision surgery is 1.5 years, varying between 0.02 and 11.0 years. In men, the average time is 0.5 years, while in women, it is 3.0 years. This fact indicates that, on average, men undergo revision surgery earlier after a femoral neck fracture compared to women. Similarly, for “Infection”, the average time to revision surgery is 3.1 years, with men undergoing surgery after an average of 2.2 years and women after 4.0 years. These comparisons provide insights into potential differences in revision surgery between men and women for different modes of hip resurfacing failure.

**Figure 3:**
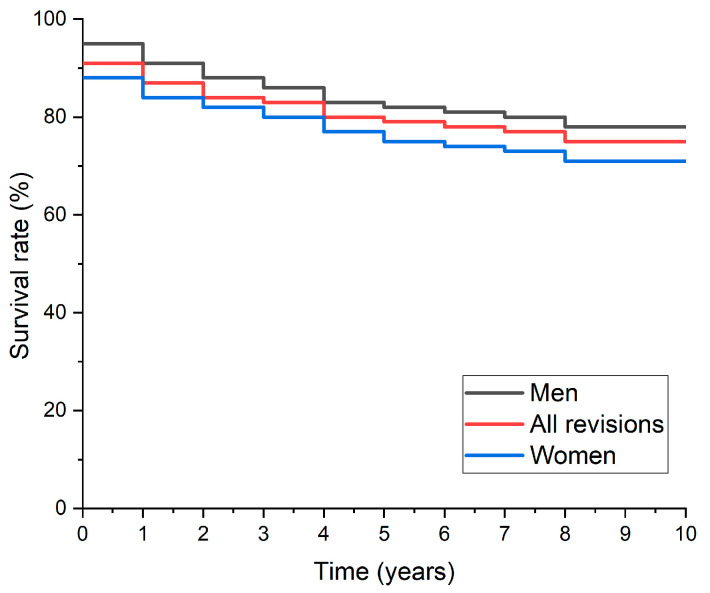
The Kaplan-Meier survival analysis depicting the Birmingham Hip Resurfacing outcomes, stratified by gender, with revision for any cause as the critical endpoint.

The 10-year survival analysis for BHR prostheses shows that the overall survival rate is 90%, with a 95% confidence interval between 85.5% and 94.5%. The results of the Kaplan-Meier survival analyses made on patients from Colentina Clinical Hospital are presented in Figure 3.

However, it is important to note that this rate may vary depending on factors such as the patient’s age and the type of complications encountered. During the 10 years of follow-up, it was observed that men had a higher survival rate (95%) compared to women (85%). This can be attributed, at least in part, to gender-specific risk factors and different anatomical features. The main reasons for failure of BHR implants during the 10 years were fracture of the femoral neck, detachment of the acetabular component, and collapse of the femoral head. Detailed analysis of failure modes, as well as a comparison between men and women for each failure mode, shows that specific problems may be more frequent or severe in certain groups of patients [48]. It has also been observed that some risk factors, such as osteoarthritis, acetabular dysplasia, and a history of previous procedures for acetabular dysplasia, can influence the stability of the acetabular component and increase the risk of implant failure [49].

Overall, this 10-year survival analysis shows that BHR implants have a good overall survival rate, although there may be significant differences between men and women in certain failure modes and average time to need surgery. It is essential for physicians to be aware of these differences and take a personalized approach to the management of patients with BHR implants.

### 2.2. Investigation Methods

Overall, in this study, various methods were used to analyze the failure of BHR surface prostheses, focusing on two main aspects: clinical aspects and explant analysis; out of a total of 300 BHR prostheses implanted, 30 experienced failures. This fact corresponds to a failure rate of 10%, as evidenced by our experimental study. To further understand the underlying causes of these failures, we conducted representative detailed case studies on 10 of these failed implants. These targeted studies provided valuable insights into the mechanical and biological factors contributing to implant failure.

**Figure 4:**
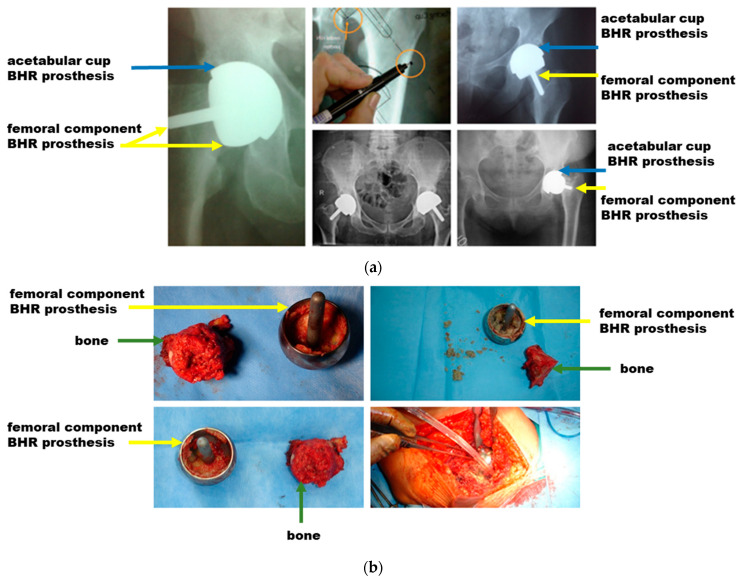
X-ray images and intraoperative analysis of BHR prosthesis failures: (**a**) X-rays showing implantation angles and assessment of joint biomechanics; (**b**) Intraoperative images illustrating the surgical technique and implantation methodology.

The first step of the analysis involved evaluating clinical aspects through X-rays and intraoperative images (Figure 4). These images were essential to assess the implantation angle and methodology used during the surgical procedure. It was found that the chosen angle and implantation technique significantly impact the prosthesis’s success, influencing the joint’s biomechanics. X-rays also allowed for a detailed analysis of how a patient’s age influences the success rate of prosthetics, revealing that patients over 50 are at higher risk of failure. This fact suggests that biomechanical factors and bone density, which change with age, are crucial to the long-term success of implants.

**Figure 5:**
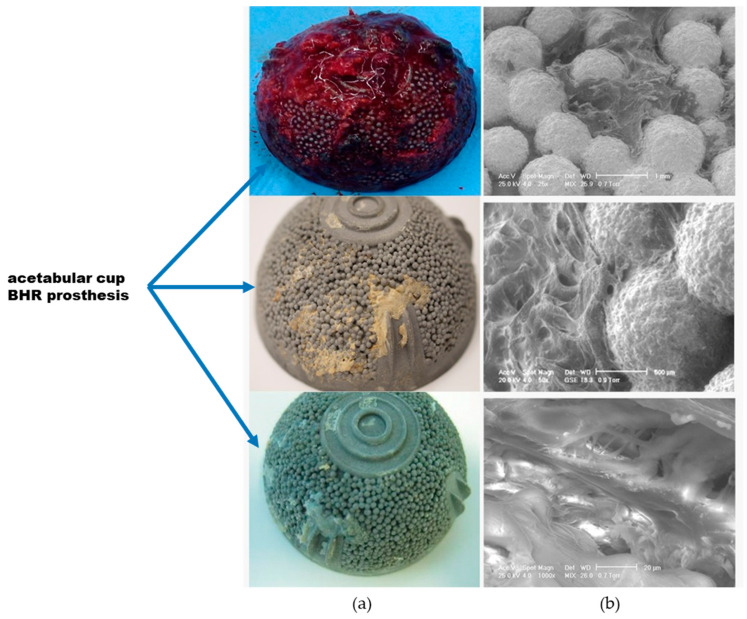
Explanted acetabular cup demonstrating successful tissue integration: (**a**) Macroscopic analysis; (**b**) Scanning electron microscopy analysis.

The second phase of the study involved detailed analysis of explants using advanced techniques such as macro examination, stereomicroscopy, and scanning electron microscopy (SEM). The macroscopic analysis was based on images taken with a Canon EOS camera, while the microscopy investigation involved a Nikon SMZ800N stereomicroscope with Koehler illumination mode (Nikon Corporation, Tokyo, Japan). This device permits image processing in NIS-Elements D software (Version 5.41.00, Nikon Corporation, Tokyo, Japan). A scanning electron microscope Philips XL 30 ESEM TMP (FEI Company, Eindhoven, The Netherlands) with low-vacuum secondary and backscattered electron detectors was used to perform very detailed analyses of the explants. We were not able to analyze all failed prostheses, but a relevant number of explants were selected for detailed analysis. The following selection criteria were considered: BHR prostheses with the same design retrieved from patient bodies between 5 and 10 years after primary surgical implantation. We excluded cases with infections and pathological fractures. Figure 5 presents an example of an explanted acetabular cup with a good osseointegration process.

## 3. Results and Discussion

Considering the data from the detailed analysis carried out in previous case studies [50,51,52,53,54,55,56,57] made on a representative sample of human subjects, we identified the most relevant issues of concern for BHR explants. These include complex issues related to the biomechanics of denture implantation, such as precision in choosing the implantation angle and impact on bone quality. The relevance of the analysis is also enhanced by the focus on BHR implants in patients over 50 years old and associated cementation methods, highlighting the imperative of a comprehensive and in-depth approach to orthopedic biomechanics.

### 3.1. Clinical Aspects Based on X-rays and Intraoperative Images

Biomechanics is the branch of science that studies the interaction between biological structures and the mechanical forces acting on them. When implanting prostheses such as BHR, the specific biomechanics of the respective joint must be considered to ensure optimal functionality. This means selecting a prosthesis that matches the patient’s natural biomechanics and positioning it to minimize joint stress and maximize the prosthesis’s stability and durability. In our study, the fundamental importance of biomechanics in the implantation process of BHR prostheses is highlighted, with special emphasis on their proper selection and positioning at the appropriate angles. X-rays and surgical images of patients requiring post-implantation revision were carefully analyzed, and it was found that incorrect positioning of prostheses leads to adverse effects. Among them is dislocation of the acetabular component, which can result in prosthesis instability, pain, and functional limitations.

**Figure 6:**
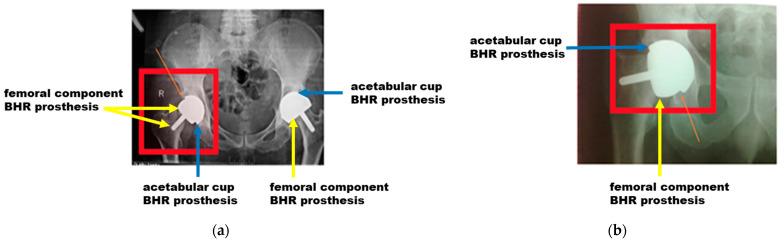
X-rays of acetabular component failure taking into consideration the edge-loading at different degrees of load: (**a**) Excessive vertical bucket positioning; (**b**) A higher degree of inclination due to an inefficient positioning maneuver.

Figure 6 shows edge-loading-induced wear attributable to excessive vertical bucket positioning. In Figure 6b, one can notice that the degree of inclination is even more pronounced, leading to progressively much more increased wear. A good understanding of biomechanics can play a role in preventing such a wear process.

**Figure 7:**
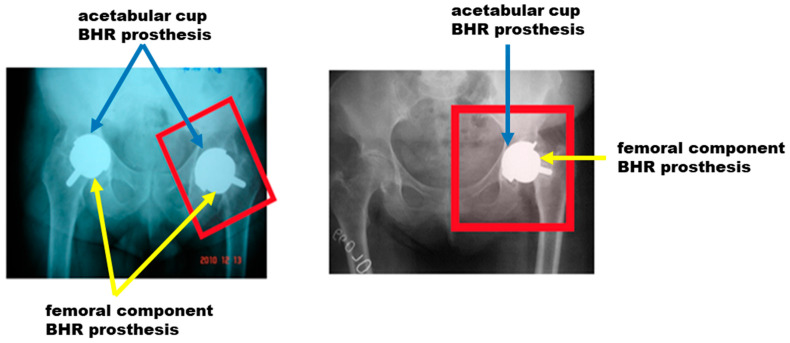
X-rays of femoral component failure: analysis of the consequences of inadequate positioning of the prosthesis.

In addition, inadequate positioning of the femoral component can lead to misalignment, asymmetric wear and tear, and the risk of bone fracture. As can be seen in Figure 7, an incorrectly chosen positioning angle caused the femoral component to move and led to a displacement of the prosthesis outside the integration area.

**Figure 8:**
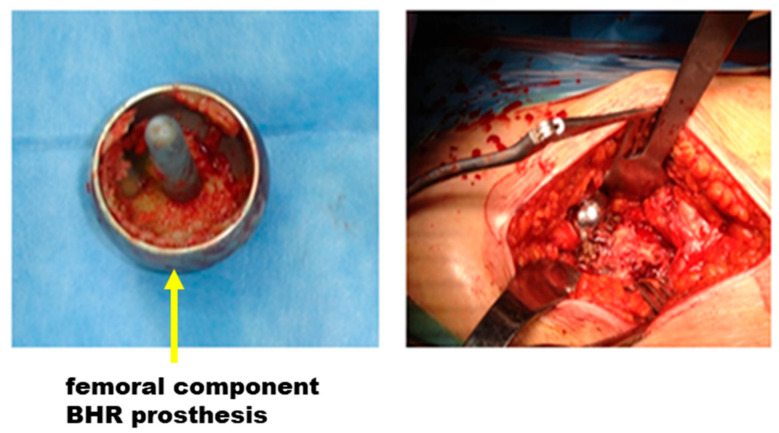
Intraoperative images: monitoring bone integration and adjusting prosthesis position.

Analysis of intraoperative images (Figure 8) reveals that, despite an apparent good integration between prosthesis and bone, incorrect positioning remains a recurring problem. This suggests the need for increased attention and expertise in the implantation process to minimize risks and maximize the success of orthopedic surgery.

### 3.2. Explant Analysis

We conducted a series of experimental research on explants to understand the concept of BHR prostheses and correctly assess their failures and causes. This study analyzed some failed hip replacements taken from Colentina Hospital in Bucharest. We focused on highlighting critical areas of the cement mantle and made detailed structural observations on these regions. The orthopedic cement belongs to the manufacturers Stryker Howmedica Osteonics and Groupe Lepine. Viscosity describes cement’s resistance to flow. Cement is classified according to the state in which it remains the longest. Typically, a low-viscosity (LV) or high-viscosity (HV) cement remains in one state for a long time. Viscosity affects how easy the cement is to handle and how well it penetrates the cancellous bone pores to achieve fixation. HV cement cannot be pressurized into bone compared to medium-viscosity cement. Table 6 provides information related to the chemical composition of the investigated orthopedic cements.

The experimental study was based on three samples, each divided into three primary focus areas used to analyze cement thickness and penetration. The cement sheath was defined as the layer of cement between the implant component and the edge of the spongy bone. Cement penetration has been characterized as the cement material that interdigitates between the surface of the reshuffled bone and deeper bone tissue.

**Figure 9:**
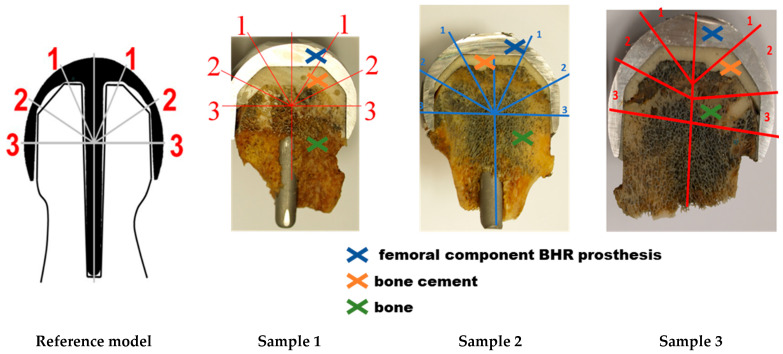
Contextual exemplification of analyzed samples according to the reference model: regions of interest used for cement thickness and penetration analysis (1–3).

Cement thickness measurements were carried out in three distinct areas: from the cap dome (zone 1), from the intermediate zone (zone 2), and from the radial zone (zone 3), on each side of the rod (Figure 9). We carefully examined the scheme of this study and made precise measurements of cement mantle thickness (Table 7) for our samples. The thickness of the cement layer was measured directly on the samples using a measuring eyepiece from Carl Zeiss AG, Oberkochen, Germany, with an accuracy of 0.1 mm.

These detailed assessments allowed us to understand cement behavior in relation to bone structures and implant components.

**Figure 10:**
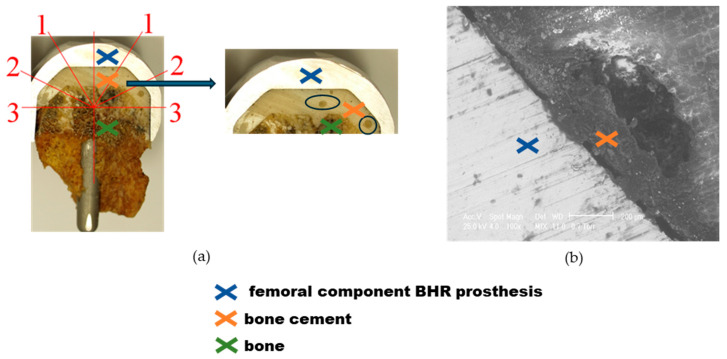
Explant BHR prosthesis—sample 1 analysis, the dome of the cap (zone 1), the intermediate (zone 2), and the radial (zone 3) regions: (**a**) Cementing defect evidenced by air bubbles; (**b**) SEM micrograph of the cement in the defect area.

Air bubbles are air spaces encapsulated in cement that occur during the mixing and application process. Following the explant presented in Figure 10a, the mixing technique used to prepare acrylic cement proved ineffective since, upon increased examination, the presence of air bubbles in the material’s mass was observed. Additionally, the viscosity obtained was unsuitable for effective application, and the cement jacket showed the same unevenness. The SEM analysis of the first sample, shown in Figure 10b, provides an enlarged image of an existing gap in the bone cement mantle. This analysis highlights critical details on the uniformity and structural integrity of the cement and reveals possible weaknesses that could compromise the long-term stability of the implant.

**Figure 11:**
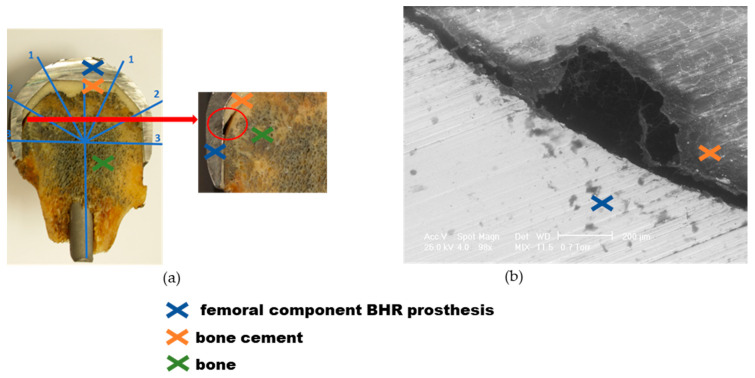
Explant BHR prosthesis—sample 2 analysis, the dome of the cap (zone 1), the intermediate (zone 2), and the radial (zone 3) regions: (**a**) Cementing defect evidenced by poor interface; (**b**) SEM micrograph of the cement in the defect area presenting crack-type defects.

Figure 11a depicts inadequate adhesion between the cement and the prosthesis surface and the adjacent bone. In Region 3, the formation of an air gap between the cement sheath and the bone is observed, which prevents the establishment of a suitable interface. Dark, porous-looking regions indicate the presence of necrotic bone, while adjacent, lighter areas highlight how cement has infiltrated the bone structure. Poor interface may be the result of an improper cement mixture or contamination with moisture or other foreign substances during the application process. The main phenomenon exposed in the case of this implant is the weakening of the prosthesis fixation, followed by its displacement and degradation over time. In addition, Figure 11b shows SEM analysis revealing a deficiency because human bone comes into direct contact with the metal part of the acetabular cup. We also note the lack of a cement-bone interface due to the space formed by improper cement application. A major defect is highlighted due to insufficient filling of the acetabular cup with bone cement.

**Figure 12:**
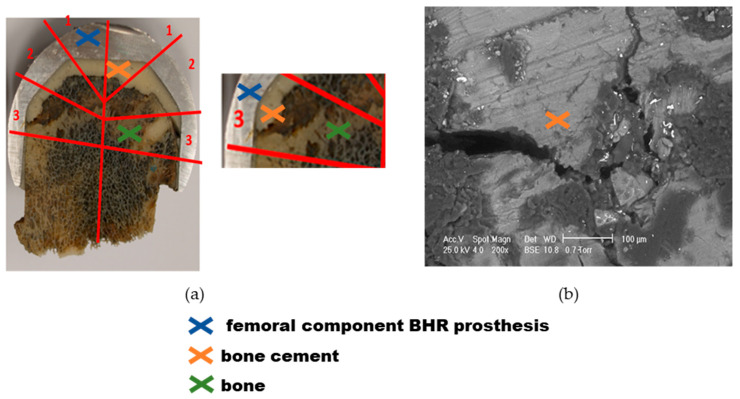
Explant BHR prosthesis—sample 3 analysis, the dome of the cap (zone 1), the intermediate (zone 2), and the radial (zone 3) regions: (**a**) Cementing defect evidenced by irregular texture; (**b**) SEM micrograph of the cement in the defect area.

Sample 3, presented in Figure 12, exhibits a more advanced stage of bone necrosis when examined macroscopically and by SEM analysis, compared to previous cases. According to the measurements of the cement mantle, we notice that it does not have the required standard dimensions and is not evenly applied. The cement penetration into the bone does not follow any rules, resulting in a gap between the cement sheath and the bone. After image magnification, it was noticed that there was a crack that started from the gap formed in the cement mantle and continued to the edges of the acetabular capsule.

### 3.3. Limitations of the Study

The study relies on retrospective analysis, which may not capture all relevant factors influencing BHR implant failure. In addition, the number of explants analyzed in the study may not represent the full range of BHR implant failure in our country. Another important limitation is that the patient population was all admitted to a single hospital, which could introduce selection bias. Our study does not include a control group for comparison, which could provide additional insights into the performance of BHR implants compared to other hip prostheses.

## 4. Conclusions

Our study on the long-term survival of Birmingham Hip Resurfacing implants showed that these devices have a 90% survival rate over 10 years, with a 95% confidence interval between 85.5% and 94.5%. However, survival varies depending on factors such as the patient’s age and the types of complications encountered.

Following a 10-year follow-up, it was observed that men had a higher survival rate (95%) compared to women (85%). This difference can be attributed to gender-specific risk factors and anatomical differences. It is overall accepted that the female gender and reduced femoral head size are directly related to an increased revision risk. Some studies [58,59] advised the practitioners not to perform hip arthroplasty in women with BHR prostheses due to the fact that women require smaller implants and the risk of failure increases directly proportional to reduced femoral head geometry [60,61]. Additionally, young women can develop dysplasia and, in some cases, much more prevalent adverse reactions to metal debris occur due to metallic implant insertion. Surgeons recommend that BHR be used for women younger than 50 years old and with a femoral head size larger than 46 mm [62,63]. From a future perspective, we will investigate the women in our selected cohort to establish exact failure cases and correlate our experimental findings with histopathological investigations.

According to the retrospective analysis of patients admitted to Colentina Clinical Hospital in Bucharest, Romania, the main causes of BHR implant failure included femoral neck fracture, detachment of the acetabular component, and collapse of the femoral head. Specific problems are more frequent or severe depending on gender and other risk factors. Risk factors such as osteoarthritis, acetabular dysplasia, and a history of previous procedures for acetabular dysplasia have been identified as significantly impacting the stability of the acetabular component and the risk of implant failure. These findings underscore the importance of a rigorous and personalized assessment of each patient before the procedure.

Our experimental research performed on the retrieved explants reveals that the main causes of BHR implant failure are consequences of other problems related to the cementation, bone quality of the patients, or malposition of the femoral component during surgery from a biomechanical point of view.

Cementing problems were identified as a major cause of failure due to the presence of air bubbles and unevenness in cement application, which compromised the adhesion and stability of the femoral component. It is important for surgeons to pay attention to the process of mixing cement components and use modern techniques such as the vacuum mixing technique.

Analysis of the most explanted acetabular components showed good osseointegration into tissue, but clinical data revealed that patients aged 50 to 60 years did not benefit from optimal implant biomechanics. The insufficient compatibility between the bone component of these patients and BHR metal-metal implants negatively influenced the long-term success of the prosthesis.

In conclusion, this study demonstrates that although BHR implants have a good long-term survival rate, their success can be affected by a number of individual factors, including age, gender, and patients’ pre-existing conditions. Physicians must consider these variables and take a personalized approach to optimize the clinical outcomes of patients with BHR implants.

## Figures and Tables

**Table 1:** Interaction between cement and bone structure.

Property	Remarks	Ref.
Adhesion	The cement must adhere well to the implant to prevent micromotion, which can lead to implant loosening. Surface treatments of implants, such as roughening or coating with bioactive materials, can enhance this adhesion	[32]
Load transfer	The cement must allow uniform mechanical load transfer between the bone and the prosthesis.	[33]
Mechanical properties	The mechanical properties of the cement must be compatible with the implant and the surrounding bone. The mechanical mismatch between the rigid PMMA and the more elastic bone can lead to stress concentrations, potentially causing microcracks and long-term failure.	[34]
Mechanical interlock	To accomplish interlock, the surface of the bones must be rough and irregular. The strength of the cement-bone interaction increases with deeper cement penetration.	[31]
Thermal effects	Because of the potential for thermal necrosis of bone tissue resulting from the exothermic reaction of PMMA curing, the ISO 5833 (2002) standard sets a 90 °C restriction on the hardening temperature of bone cement. Controlling the polymerization temperature is crucial to mitigate these effects.	[31,36]
Biocompatibility	PMMA is biocompatible, but it can cause a mild inflammatory response. The exothermic polymerization process can determine thermal necrosis. The presence of residual monomer can be cytotoxic.	[32]
Osseointegration	Osseointegration relies on mechanical interlock rather than chemical bonding. While PMMA itself is not osteoinductive, the surface texture and porosity can be modified to enhance integration with bone.	[35]

**Table 2:** Number of surgical cases for each type of medical indication, reported by patient gender.

Gender	Diagnosis								Total
	P.C.	N.A.C.F.	S.C.	D.S.P.	B.C.	O.F.H.	O.D.	P.T.	
**Women**	25	53	3	16	0	0	0	1	98
**Men**	44	122	12	21	1	1	1	0	202
**Total**	69	175	15	37	1	1	1	1	300

P.C.: primary coxitis; N.A.C.F.: aseptic necrosis of the femoral head; S.C.: secondary coxitis post-traumatic; D.S.P.: dysplasia; B.C.: bilateral coxitis; O.F.H.: osteonecrosis of the femoral head; O.D.: osteochondritis dissecans; P.T.: polyarthritis.

**Table 3:** Gender distribution across failure modes.

Mode of Failure	Total (%)	Men	Women	*p*-Estimated Value
All revisions	30 (10.0)	20	10	<0.001
Fracture of the femoral neck	9 (3.0)	6	3	0.005
Loosening of the acetabular component	6 (2.0)	4	2	0.02
Head collapse	5 (1.7)	3	2	0.03
Femoral loosening	3 (1.0)	2	1	0.06
Infection	3 (1.0)	2	1	0.06
Pain/(ALVAL)/metalloids	2 (0.7)	1	1	0.11
Malposition of the acetabular component	1 (0.3)	1	0	0.27
Dislocation	2 (0.7)	1	1	0.1

**Table 4:** Pre-operative diagnoses for acetabular component loosening revisions.

Preoperative Diagnosis	Men	Women	Potential Adverse Effect on Acetabular Component Stability
Osteoarthritis	10	5	No
Acetabular Dysplasia	1	1	Yes
Previous Shelf Procedure for Acetabular Dysplasia	1	1	Yes
Renal Failure	1	1	Yes
Avascular Necrosis of the Femoral Head Following Radiotherapy for Pelvic Osteosarcoma	1	1	Yes
Unknown	3	2	Unknown

**Table 5:** Comparison of mean time to revision surgery for different modes of failure between men and women.

Mode of Failure	Total (%)	Men	Women	*p* Estimated Value
All revisions	2.9 (0.003 ÷ 11.0)	1.7 (0.04 ÷ 8.2)	4.5 (0.002 ÷ 11.0)	<0.001
Fracture of the femoral neck	1.5 (0.02 ÷ 11.0)	0.5 (0.04 ÷ 3.8)	3.0 (0.002 ÷ 11.0)	0.0012
Loosening of the acetabular component	2.4 (0.01 ÷ 9.7)	1.4 (0.04 ÷ 3.0)	3.0 (0.01 ÷ 9.7)	0.16
Head collapse	3.9 (0.3 ÷ 9.8)	3.1 (0.6 ÷ 7.8)	5.2 (0.3 ÷ 9.8)	0.28
Femoral loosening	3.7 (0.1 ÷ 9.1)	3.0 (0.1 ÷ 5.8)	4.4 (0.4 ÷ 8.8)	0.50
Infection	3.1 (0.5 ÷ 9.6)	2.2 (0.5 ÷ 4.4)	4.0 (1.0 ÷ 9.6)	0.53
Pain/ALVAL/metalloids	5.2 (1.0 ÷ 10.2)	3.3 (1.0 ÷ 6.5)	6.8 (1.7 ÷ 10.2)	0.16
Malposition of the acetabular component	4.2 (1.5 ÷ 6.5)	1.5 (N/A)	6.0 (1.5 ÷ 6.5)	N/A
Dislocation	3.9 (0.003 ÷ 9.5)	0	3.9 (0.003 ÷ 9.5)	N/A
Loosening of both components	3.4 (0.3 to 5.9)	1.6 (0.3 to 3.0)	4.6 (3.4 to 5.9)	1.0

**Table 6:** Chemical composition of the bone cement samples used in our study [30]. Table is licensed under CC-BY-4.0.

		Surgical Simplex P (Stryker Howmedica Osteonics)	Aminofix 1 (Groupe Lepine)	Aminofix 3 (Groupe Lepine)
**Liquid component**		**18.79 g**	**14.4 g**	**16.4 g**
Methyl methacrylate	Monomer	18.33	12.28	13.99
Butyl methacrylate	Monomer	-	1.90	2.16
N,N-dimethyl-p-toluidine	Activator	0.46	0.22	0.25
Hydroquinone	Inhibitor	60 ppm	20 ppm	20 ppm
**Powder component**		**41 g**	**40 g**	**40 g**
Polymethyl methacrylate	Pre-polymerized polymer	6.00	33.68	33.52
Benzoyl peroxide	Initiator	0.50	0.96	1.12
Methyl methacrylate-styrene copolymer	Pre-polymerized copolymer	30.00	-	-
Barium sulphate	Radiopaque agent	4	3.84	3.84
Gentamicin sulfate	Antibiotic	-	1.52	1.52
Erytromycin Colistin	Antibiotic	0.50	-	-
Sulphomethate	Antibiotic	3.00 million I.U.	-	-
Sodium EP viscosity		low	standard	low

**Table 7:** Evaluations of contact areas between the acetabular cup and the acrylic cement mantle.

Sample	Zone	Average Thickness of the Cement Layer (mm)
Sample 1	1	24.86
	2	15.68
	3	13.92
Sample 2		12.83
	2	7.59
	3	5.90
Sample 3	1	13.48
	2	12.29
	3	9.43

## Data Availability

The original contributions presented in the study are included in the article, further inquiries can be directed to the corresponding author.
